# The need for speed? Examples from a randomised controlled trial in the emergency setting

**DOI:** 10.1186/1745-6215-14-S1-P130

**Published:** 2013-11-29

**Authors:** Seonaidh Cotton, Tracey Davidson, Nishat Siddiqi, Michael Frenneaux

**Affiliations:** 1 University of Aberdeen, Aberdeen, UK

## 

There are a number of unique challenges associated with setting-up and running randomised controlled trials in an emergency setting.

Within the NIAMI (Nitrates in Acute Myocardial Infarction) Trial patients present as an emergency and clinicians aim to start primary percutaneous coronary intervention (PPCI) without unnecessary delay. The trial drug is delivered before PPCI is commenced - so there is some urgency to assess the patient's eligibility, gain agreement from the patient to take part in the trial and deliver the trial drug. To minimise any delay in commencing PPCI, we designed the study so that the inclusion and exclusion criteria are simple and quick to assess, patients are given a short verbal explanation of the study and give verbal agreement if they wish to take part (we seek fully informed consent 6-48 hours later), and the study drug packs are pre-randomised and stored in the facility where PPCI is undertaken (to minimise any delay in using telephone or web-based randomisation and collecting the study drug from Clinical Trials Pharmacy). To meet recruitment targets, 24-hour recruitment was implemented - this relied on the goodwill and buy-in from the entire clinical team (and not just from the immediate trial team who were often not present out-of-hours). Overcoming these challenges at the design and implementation phases of the trial has contributed to it reaching recruitment targets.

Drawing on our experience from NIAMI, we will discuss some of the challenges, and how these were overcome.

